# Automated Landslides Detection for Mountain Cities Using Multi-Temporal Remote Sensing Imagery

**DOI:** 10.3390/s18030821

**Published:** 2018-03-09

**Authors:** Zhong Chen, Yifei Zhang, Chao Ouyang, Feng Zhang, Jie Ma

**Affiliations:** National Key Laboratory of Science & Technology on Multi-Spectral Information Processing, Huazhong University of Science and Technology, 430074 Wuhan, China; henpacked@163.com (Z.C.); ouyangchao16@hust.edu.cn (C.O.); M201572329@hust.edu.cn (F.Z.); majie@mail.hust.edu.cn (J.M.)

**Keywords:** landslides detection, remote sensing images, change detection, Deep Convolution Neural Network, Spatial Temporal Context Learning

## Abstract

Landslides that take place in mountain cities tend to cause huge casualties and economic losses, and a precise survey of landslide areas is a critical task for disaster emergency. However, because of the complicated appearance of the nature, it is difficult to find a spatial regularity that only relates to landslides, thus landslides detection based on only spatial information or artificial features usually performs poorly. In this paper, an automated landslides detection approach that is aiming at mountain cities has been proposed based on pre- and post-event remote sensing images, it mainly utilizes the knowledge of landslide-related surface covering changes, and makes full use of the temporal and spatial information. A change detection method using Deep Convolution Neural Network (DCNN) was introduced to extract the areas where drastic alterations have taken place; then, focusing on the changed areas, the Spatial Temporal Context Learning (STCL) was conducted to identify the landslides areas; finally, we use slope degree which is derived from digital elevation model (DEM) to make the result more reliable, and the change of DEM is used for making the detected areas more complete. The approach was applied to detecting the landslides in Shenzhen, Zhouqu County and Beichuan County in China, and a quantitative accuracy assessment has been taken. The assessment indicates that this approach can guarantee less commission error of landslide areal extent which is below 17.6% and achieves a quality percentage above 61.1%, and for landslide areas, the detection percentage is also competitive, the experimental results proves the feasibility and accuracy of the proposed approach for the detection landslides in mountain cities.

## 1. Introduction

Landslides are a major hazard in lots of countries, causing huge property losses and many casualties every year [1]. Especially in the areas that are close to both cities and mountains, landslides that are triggered by earthquakes or heavy rain happen frequently and cause even more damage, just like those that happened in Wenchuan [2]. Against this background, quick landslides hazard emergency response and accurate risk assessment based on landslide inventories are of great importance and the key of them is to detect critical areas [3,4]. If a landslide area is detected, its specific analysis can be carried out for emergency response and the precautions can be done.

Remote sensing is an ideal tool for landslides detection because it offers a wide area of territorial data with various degrees of both spatial and temporal resolution. It has been widely used in landslides detection with the help of image analysis technology, which can save human resources costs and make the process more automatic. In the past, there are two main approaches that use remote sensing images in landslides detection, one of them only makes use of the post-event data and the landslides areas are identified by classification, such as scene classification method [5], object-oriented analysis and classification method [6,7], pixel-based classifying method using Bayesian framework [8], generalized positive Boolean function [9], and neural network [10]. The methods using post-event image for classification assume that all the landslides are triggered by the same event like earthquake, rainstorm, and so on, and have similar spectral and texture characteristics, without indicating the time period when the landslides happened.

The other one uses data of pixels or features that are calculated by rule to conduct multi-temporary change detection between pre-event and post-event data, including the method based on image difference and landslide related factor [11], method based on change detection [12], method using change detection coupled with false alarm removal [13] or texture analysis [14], and so on. Binary temporal methods can use temporal spatial information to reduce the search area and specify the time period that is determined by the dates of experimental image pair.

Both of the two approaches use classification to confirm the final landslide areas, the classification is conducted based on the model trained by landslide samples [5,9,10,15] or the rules made of features selected by experts [6,7,8,11,12,13,14,16], the parameters of model are determined through trial and error, and the threshold of each feature is determined through the knowledge of landslide areas that have been found. Actually, the use of classification inevitably leads to omission/commission errors, and applying artificial features to recognition landslides would mistake other land covering types for landslides because the spatial regularity that only relates to landslides have not be found. Additionally, most methods using training samples are semi-automated because samples should be extracted by experts and it is difficult to acquire appropriate landslide samples in many areas where there is no landslides that have recorded in the past. For the above reasons, an automated method, which uses landslide-related knowledge for landslides detection without information of landslides inventories, is needed. Behling et al. [17] have proposed a multi-temporal landslide detection method based on NDVI-trajectories and relief-oriented analysis because the occurrence of landslides may cause an abrupt decrease of vegetation covering in mountainous regions. However, the method using only the change of vegetation covering can not identify the whole landslide areas in mountain cities where the occurrence of landslides may cause more casualties and serious economic losses, because landslides in mountains cities may cause not only the decrease of vegetation covering, but also the vanishing of buildings.

Deep Convolutional Neural Network (DCNN) have attracted widespread is the element-wise product. Then, the spatial context model can be calculated byattention since Krizhevsky et al. [18] revealed its outstanding capacity for image classification in 2012, and then it was successfully applied to object detection [19], natural language processing [20], and speech recognition [21]. The multi-layer feature extraction framework of DCNN contributes to abstracting the features that can more effectively describe the nature of images than artificial features, because artificial features usually only concentrate on one aspect of spatial regularity, such as texture or spectrum. Spatial Temporal Context Learning (STCL) was proposed by Zhang et al. [22] for accurately real time visual tracking. The method mainly builds spatial context model of object and uses the model for detection in time-series images. When compared with the classification method, STCL makes full use of the temporal and spatial information, and avoids omission/commission errors, thus it was chosen for landslides detection.

In this article, a novel automated method based on the knowledge about landslide-related surface covering change in mountain cities is proposed. The approach introduces a new change detection method based on DCNN to find changed area. The STCL is used to identify the specific landslide areas based on the change of vegetation covering and built-up areas. The vegetation covering areas are represented by Normalized Difference Vegetation Index (NDVI), and the built-up areas are identified by calculation of the texture-derived built-up presence index, which is called PANTEX [23]. The feasibility and accuracy of the proposed method is validated using remote sensing imagery of mountain cities.

## 2. Study Area and Data

Landslides in Shenzhen, Zhouqu Country, and Beichuan County are chosen to validate the proposed method. These three Chinese cities are mountainous and they suffered significant losses because of landslides. The landslides of Shenzhen dated on 20 December 2015 led to the loss of 69 lives and dozens of buildings in ruins, and the massive landslides coupled with debris flow in Zhouqu County where earthquakes, landslides, and debris flow often occur, happened on 7 August 2010, leaving 1765 people dead or missing, and smashed thousands of houses. Beichuan County was devastated because of the Wenchuan earthquake happened on 12 May 2008, the earthquake triggered lots of landslides that destroyed the city and buried thousands of people. 

**Figure 1:**
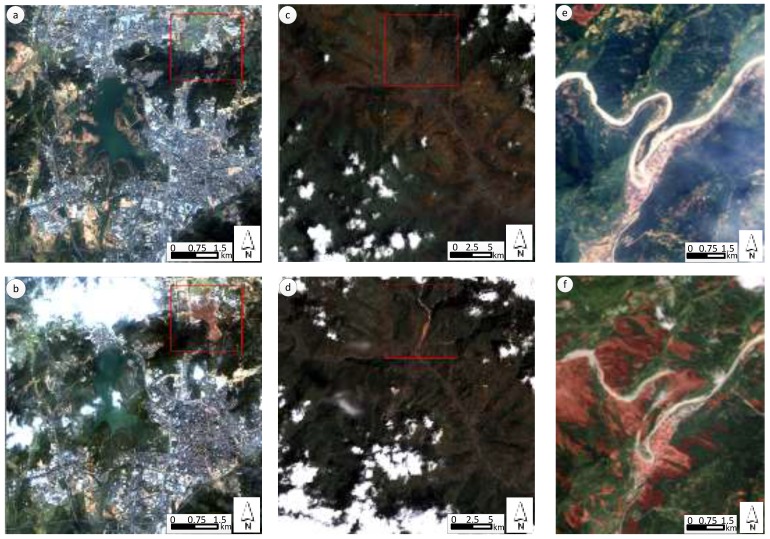
Pre- and post-event satellite imagery. (**a**) GF-1 data taken in Shenzhen, China on 24 December 2013; (**b**) GF-1 data taken in Shenzhen, China on 29 December 2015; (**c**) Landsat-8 data taken in Zhouqu County, China on 13 July 2009; (**d**) Landsat-8 data taken in Zhouqu County, China on 10 September 2010; (**e**) Formosat-2 data taken in Beichuan County on 5 April 2006; (**f**) Formosat-2 data taken in Beichuan County on 16 May 2008. All of above images have 4 bands (blue, green, red and near infrared). The landslides in Beichuan County are different from the rest because of the wide coverage and the large sum.

**Figure 2:**
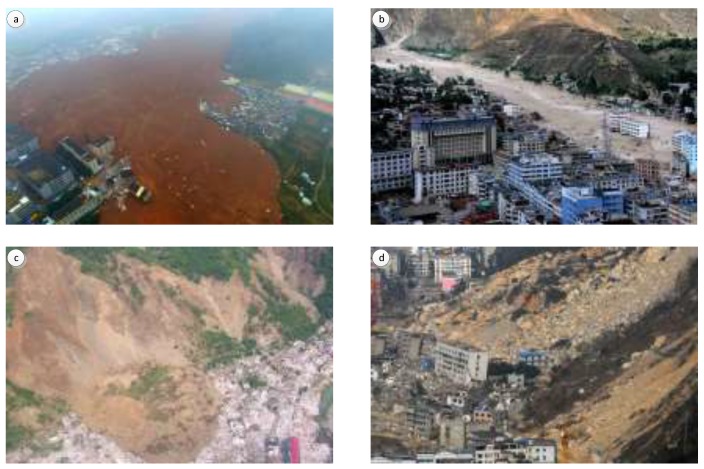
(**a**) Aerial images of landslide regions in Shenzhen; (**b**) Aerial images of landslide regions in Zhouqu County; (**c**,**d**) Aerial images of landslide regions in Beichuan County.

Figure 1 shows the experimental images for landslides detection. The three image sets are processed with registration [24], and then histogram matching is applied to removing the influence of discrepant radiation intensity [25]. Landslides that had taken place in the three sites led to drastic alteration in land surface and obvious sediment transport. In Figure 2a–d, we can see the sediment transport bury vegetation and buildings. Additionally, the landslides detection method uses a digital elevation model (DEM) of 5 m spatial resolution to derive slope information, which can make the landslides detection more reliable [17], and the change of DEM is also used for recognizing the residual part of landslide areas where there are no change of the cover type. 

## 3. Methodology

**Figure 3:**
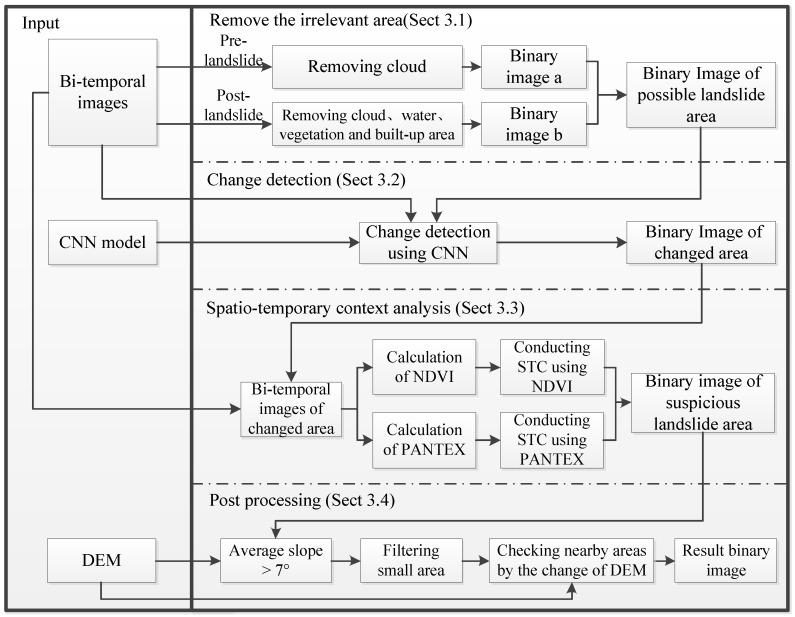
Integral process of proposed landslides detection approach for mountain cities using multi-temporal remote sensing imagery.

The proposed method aims at the automated landslides detection in mountain cities using multi-temporal remote sensing data. Thus, the changes of the earth surface during the analyzed time span should be taken in consideration and the landslide-related phenomena in the study area should be used for extracting the landslide areas. To meet these requirements, the method uses a patch-oriented change detection and conducts a spatial-temporal analysis of the NDVI and PANTEX in the changed patches of the image. The Integral process of the proposed method is showed in Figure 3.

Procedures of the proposed approach for landslides detection are as following:

1. Cloud-covered regions ought to be abandoned in all images. Unconcerned regions with vegetation, water and building are also supposed to be discarded in the post-event image, then the possible landslide areas are obtained (Section 3.1).
2. Extract changed areas by applying change detection with Deep Convolution Neural Network to pre- and post-event images (Section 3.2).
3. STCL aiming at binary temporal NDVI and PANTEX in changed areas is used and the suspicious landslide areas is extracted (Section 3.3).
4. The post process is added to get more reliable and complete landslide areas (Section 3.4).

### 3.1. Remove the Irrelevant Areas

According to the terrain transformation analysis [26] that is related to landslides, regions that are covered by vegetation, water, and buildings in post-event images can be ignored to reduce the area of terrain to be detected, and the computational cost of the whole method. Besides, areas with cloud in pre- and post-landslide images should be abandoned to guarantee finding real changed area in change detection.

The built-up areas are extracted by calculating PANTEX, the index is based on fuzzy rule-based composition of anisotropic textural co-occurrence measures that are derived from the gray-level co-occurrence matrix (GLCM) [23]. Calculating NDVI, Normalized Difference Water Index (NDWI), for each pixel is a simple and quick method to obtain vegetation covering and water covering from remote sensing data [27,28]. The two indexes are defined as

(1) $$\mathrm{NDVI}=\frac{N_{\mathrm{IR}}-R}{N_{\mathrm{IR}}+R},$$

(2) $$\mathrm{NDWI}=\frac{G-N_{\mathrm{IR}}}{G+N_{\mathrm{IR}}},$$

where $N_{\mathrm{IR}}$ stands for near infrared band, R stands for red band and G is the green band.

In the experiment, the threshold of NDVI and NDWI is 0.32 and 0, respectively, the pixel whose value is higher than the threshold means that there is vegetation or water, and the pixel whose PANTEX is higher than 0.3 would be determined as a built-up area. For masking clouds, a threshold (threshold = 180) has been applied to the average value of four bands, the threshold is selected thinking of separating temporal bright objects (e.g., clouds) from permanent ones (e.g., buildings and sand).

After marking the vegetation, water, and built-up areas in post-event images and clouds covering in all of the images, binary image a and b in Figure 3 can be generated. The values of pixels in binary image a would be 0 if the pixel in the same position in pre-landslide image have been marked; other pixels in binary image a would take 1 as their value. For binary image b, the values of pixels which have been marked in post-landslide images would be 0, others would be 1. Binary image that shows possible landslide regions is made of above images a and b, it is the result of “and” operation from two images, so its pixel whose value is 1 represents the possible landslide region.

### 3.2. Change Detection Using DCNN

**Figure 4:**
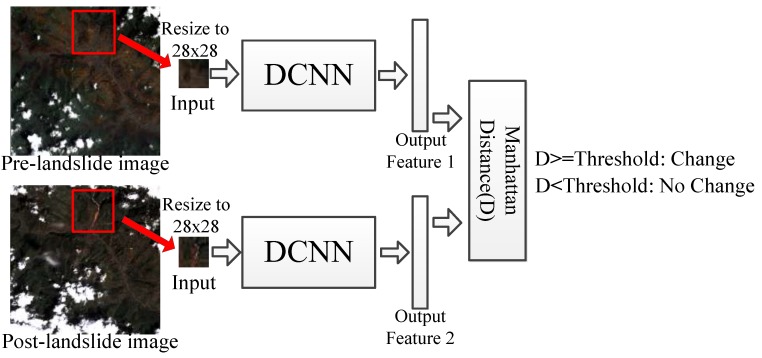
Change detection based on Deep Convolution Neural Network.

In this section, change detection is applied to the possible landslide areas that have been found in Section 3.1. As shown in Figure 4, the key ideas are leveraging the high capacity of Deep Convolutional Neural Network for feature extraction [29,30] to learn robust representations of images, and calculating the Manhattan distance between representations of corresponding possible landslide area in two images, the Manhattan distance is used for measuring the change degree, and then a threshold is used to determine whether the change have occurred in this area.

#### 3.2.1. Feature Extraction

**Figure 5:**
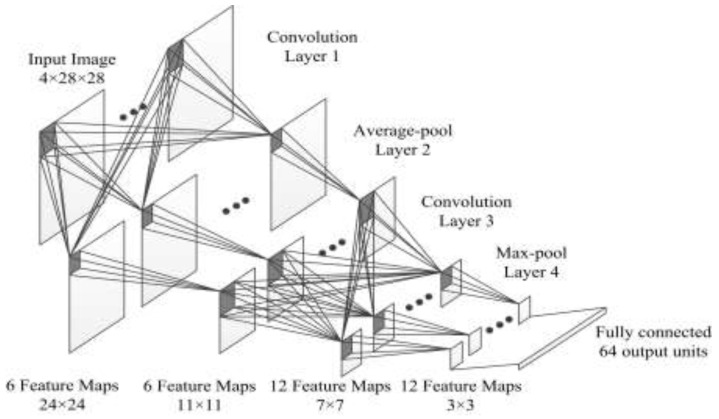
Structure of the Deep Convolution Neural Network.

The architecture of our DCNN model is summarized in Figure 5. It contains five learned layers—two convolutional, two pooling (average pooling and max pooling), and a fully connected. In convolutional process, the size of kernels is 5 × 5 and the stride parameter is 1; in pooling process, the size of kernels is 3 × 3 and the stride parameter is 2. The DCNN model is trained by SAT-4 [31] dataset, and an extra fully connected layer is added for classification in the training process, this layer is not shown in Figure 5 because it would be abandoned in the feature extraction process.

After the DCNN model has been learnt, the possible landslide pixels with their 100 × 100 neighbor would be resized to 28 × 28 image patches that are the input of the model, and the 64-D output vector is the feature of corresponding image patch. Due to the multilayer deeply abstraction of DCNN, the feature consists of spectrum, texture, and shape information that can describe the nature of the input image patch.

#### 3.2.2. Change Detection

Since the feature extracted by DCNN can describe the nature of the image, if changes happen in an area, the feature of the corresponding part of the image would change accordingly. Therefore, after extracting the two features of the corresponding 100 × 100 neighbor that center on the same possible landslide pixel in pre- and post-landslide image, the Manhattan distance is calculated between these two features. Manhattan distance between feature ($x_{1},x_{2},\ldots ,x_{n}$) and feature ($y_{1},\;y_{2},\;\ldots ,y_{n}$) can be expressed as follows:

(3) $$D=\;\left|x_{1}-y_{1}\right|+\left|x_{2}-y_{2}\right|+\ldots +\left|x_{n}-y_{n}\right|,$$

**Figure 6:**
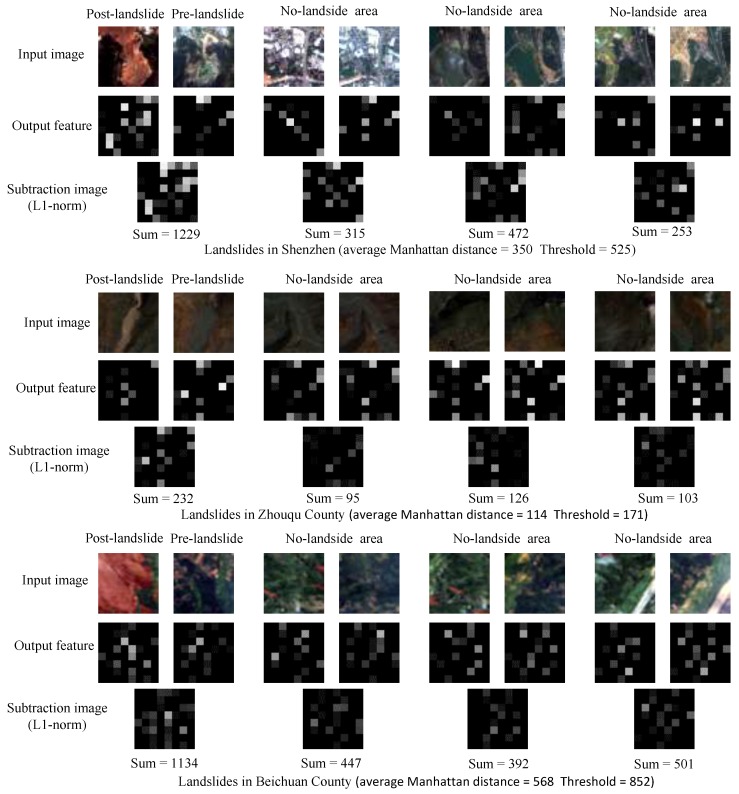
Visualizing the change detection process of three landslides. For each landslides, the first row images are 4 pairs of image patches chosen for visualization, each pair image patches are the same area in pre- and post-landslide image, the first pair is landslide area, others are no-landslide area; The second row are output features of DCNN, the 64-D output features are reshaped to 8×8 feature images for visualization; Subtraction operator is conduct on each pair of feature images with L-1 norm, the results are shown in the third row. The sum of pixels values in every subtraction image is calculated, it is equal to Manhattan distance between the two features of the pair of image patches.

If the Manhattan distance is bigger than the threshold, the pixel and its 100 × 100 neighbor would be determined as a changed possible landslide area, and the value of pixels in this area can be retained based on the binary image that shows possible landslide regions in Section 3.1; but when the Manhattan distance does not reach the threshold, these pixels would be modified to 0. Though the threshold should be modified when the experimental area or data is changed, it is not sensitive and it can be set to 1.5 times the average Manhattan distance between features of all the corresponding patches in pre- and post-landslide image. The process is visualized in Figure 6. It obviously shows that the landslide-related surface covering change causes larger variation of features than that caused by common artificial or natural surface covering change. 

After this procedure, a binary image that indicates changed regions have been formed. Because the change detection is only applied to possible landslide pixels and their neighbor, the pixels whose values are still 1 represent the areas where the landslide may occur and the striking changes of the earth surface have happened between the date of pre- and post-landslide image.

### 3.3. Spatial Temporal Context Analysis

In this section, the exact landslide areas are detected based on spatial and temporary information, and the process is pixel-oriented for higher extraction accuracy. The STCL algorithm is used in this process, it is based on formulating the spatial and temporal relationships between the target and its local context. A Bayesian framework is used to express the statistical correlation of low-level features (i.e., intensity and position). In our research, changed pixels in Section 3.2 are regarded as the target and the two type spatial context models of these pixels are built based on NVDI and PANTEX information, respectively, in pre-landslide image, then the NDVI models and PANTEX models are used to identify the same position in the post-landslide image to determine weather the vegetation or built-up area have disappeared, in consideration of the landslide-related disappearance of vegetation and buildings in mountain cities.

#### 3.3.1. Spatial Context Modeling

In STCL, a confidence map is computed as a following equation to estimate the target location likelihood:

(4) $$\begin{array}{ll} c(x) & =P(x|o)\\ & =\sum_{c(z)\in X^{c}}P(x|c(z),o)P(c(z)|o), \end{array}$$

where $x\in {\mathbb{R}}^{2}$ is the location of target and o indicates the target present in the scene. The spatial context set is defined as $X^{c}=\{c(z)=(I(z),z)|z\in \Omega_{c}(x^{*})\}$ and I(z) denotes the intensity of location z in image, $\Omega_{c}(x^{*})$ denotes the 21×21 neighborhood of the object location $x^{*}$. Location likelihood is based on condition probability P(x|c(z),o) and prior probability P(c(z)|o). The condition probability is spatial context model that describes the spatial relationship between the target and its context information, it is defined as:

(5) $$P(x|c(z),o)=h^{sc}(x-z),$$

where $h^{sc}(x-z)$ is related to the distance and direction between the location x of the target and its neighbor context location z. P(c(z)|o) denotes a context prior probability, it is computed as follows:

(6) $$P(c(z)|o)=I(z)w_{\sigma }(z-x^{*}),$$

with a weighted function $w_{\sigma }(x)$ which is defined by

(7) $$w_{\sigma }(x)=a\times \exp[-|x{|}^{2}/\;(\sigma^{2})],$$

where parameter a is used for normalization and σ is a scale parameter.

If the target location $x^{*}$ is known, the confidence map can be calculated as the following equation:

(8) $$c(x)=P(x|o)=b\times \exp[-|(x-x^{*})/\;\alpha {|}^{\beta }],$$

where *b* is a normalization constant, β and α is, respectively, scale parameter and shape parameter.

Based on (5)–(8), the likelihood (4) can be written as:

(9) $$\begin{array}{ll} c(x) & =b\times \exp[-|(x-x^{*})/\;\alpha {|}^{\beta }]\\ & =\sum_{z\in \Omega_{c}(x^{*})}h^{sc}(x-z)I(z)w_{\sigma }(z-x^{*})\\ & =h_{}^{sc}(x)⊗(I(x)w_{\sigma }(x-x^{*})) \end{array},$$

where ⊗ represents the operator of convolution.

In consideration of that convolution can be replaced by Fast Fourier Transform (FFT), (8) can be expressed as:

(10) $$ℱ(b\times \exp[-|(x-x^{*})/\alpha {|}^{\beta }])=ℱ(h^{sc}(x))\;⊙\;ℱ(I(x{)w}_{\sigma }(x-x^{*})),$$

where ℱ denotes FFT and ⊙ is the element-wise product. Then, the spatial context model can be calculated by

(11) $$h^{sc}(x)={ℱ}^{-1}\left\{\frac{ℱ(b\;\times \;\exp[-|(x\;-\;x^{*})/\alpha {|}^{\beta }])}{ℱ(I(x{)w}_{\sigma }(x\;-\;x^{*}))}\right\},$$

where ${ℱ}^{-1}$ is the inverse FFT.

In our experiment, every changed pixel in possible landslide area is regarded as target for which two models ($h_{NDVI}^{sc}(x)$ and $h_{PANTEX}^{sc}(x)$) are built, $h_{NDVI}^{sc}(x)$ is based on NVDI image computed as (1), $h_{PANTEX}^{sc}(x)$ is based on PANTEX image computed as [23], the parameters of likelihood function are set to α=2.25 and β=1, the scale parameter σ=1 and the normalization constant a and b is respectively 0.075 and 0.5 according to [22].

#### 3.3.2. Temporary Confidence Learning

After getting the spatial context model of the changed possible landslide pixel in pre-landslide image, a confidence is computed for the same location in post-landslide image. At first, the NDVI image and PANTEX image are calculated based on post-landslide image, and then for every changed possible landslide pixel, convolution process using NDVI spatial model and PANTEX spatial model is conducted to compute the confidence maps, as follows:

(12) $$c_{NDVI}(x)={ℱ}^{-1}(ℱ(h_{NDVI}^{sc}(x))\;⊙\;ℱ(I_{NDVI}{(x)w}_{\sigma }(x-x^{*}))),$$

(13) $$c_{PANTEX}(x)={ℱ}^{-1}(ℱ(h_{PANTEX}^{sc}(x))\;⊙\;ℱ(I_{PANTEX}{(x)w}_{\sigma }(x-x^{*}))),$$

where $I_{NDVI}(x)$ and $I_{PANTEX}(x)$ denotes the intensity of locating x in NDVI image and PANTEX image calculated based on post-landslide image, $x\in \Omega_{c}(x^{*})$, where $x^{*}$ is the location of the changed possible landslide pixel. The confidence map is a 21×21 matrix, and the likelihood L of the location in post-landslide image is the value of matrix center, L is represented as $L_{NDVI}=c_{NDVI}(11,11)$, and $L_{PANTEX}=c_{PANTEX}(11,11)$. if $L_{NDVI}$ or $L_{PANTEX}$ is smaller than 0, it seems that changes have happened in the vegetation or built-up area, and the corresponding pixel is determined as suspicious landslide pixel because landslides can lead to disappearing of vegetation and buildings in mountain cities.

### 3.4. Post Processing

Suspicious landslide areas are further evaluated with relief-based plausibility using the slope parameter which is derived from DEM and has been generally used for landslide validation [32,33,34], it considers the fact that the occurrence of landslides needs an initial relief contrast that enable the downward flow of material. In the experiments, the suspicious landslide areas whose average slope is greater than 7° are confirmed to be landslide areas. Then, minute and scattered detected regions are supposed to be filtered out according to their size, because of the spatial concentration of landslides, and the size threshold is 50 (pixels) in the experiments. Finally, because there are some areas without cover type changes but with topography changes should also be part of landslide areas, and they are usually connected to landslide areas that are with cover type changes, thus for all of the pixels that are connected to the detected landslide areas, the change of DEM data in corresponding position should be taken into account. If the change of DEM data is higher than 4m, then the corresponding pixel would be taken as part of landslide areas, and the pixels that are connected to this new part of landslide areas should also be checked in the same way. 

## 4. Result and Discussion

### 4.1. Experiment Result

**Figure 7:**
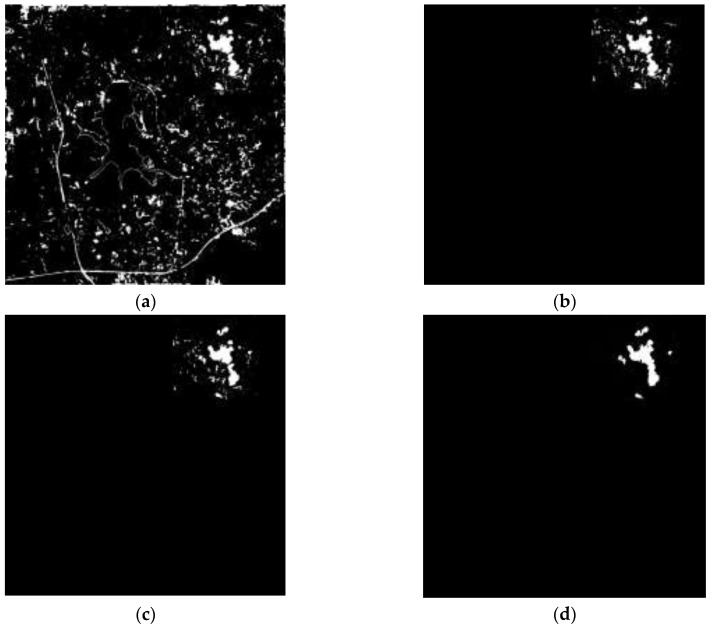
Experimental results of Shenzhen, China. (**a**) Black-white image of possible landslide regions (Section 3.1); (**b**) Black-white image of changed regions (Section 3.2); (**c**) Black-white image of suspicious landslide regions (Section 3.3); and, (**d**) Final result image (Section 3.4).

Figure 7 shows the results of Shenzhen’s landslide areas being detected by the proposed method. The binary image of possible landslide areas is shown in Figure 7a, from which we can see the possible landslide areas, which are represented by white pixels, occupy a small number of pixels and have very high concentration in landslide area because the main landslide-unrelated land covering types (i.e., vegetation, water, and built-up area) have been extracted and removed. Figure 7b shows changed possible landslide areas that are marked as white pixels, when compared to Figure 7a, this process only retains the white pixels that are in changed area, and removes others to prevent the confusing causing by land covering types that are visually similar to landslides in remote sensing images (e.g., bare land). In Figure 7c, we can see some white regions were abandoned by STCL because in the changed area there are still many areas that are visually similar to landslide. With the help of STCL that is based on NDVI and PANTEX, only the areas where vegetation or buildings have disappeared in the given period can be left, in keeping with the knowledge about landslide-related surface covering changes in mountain cities. In consideration of the concentrative and spatial pattern of landslides, regions that are smaller than 50 are filtered out, and some pixels which are connected to the detected landslide areas are included as part of landslide areas because of the great change of DEM in corresponding areas, the final result is shown in Figure 7d.

**Figure 8:**
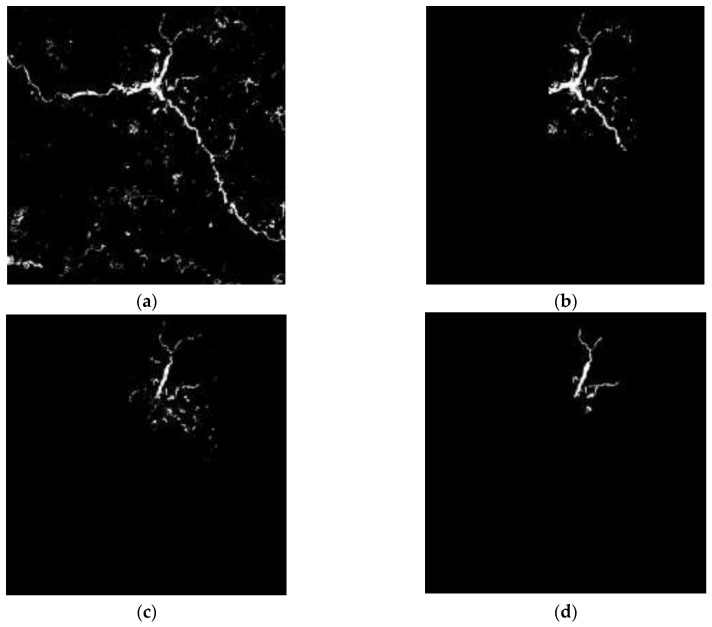
Experimental results of Zhouqu County, China. (**a**) Black-white image of possible landslide regions (Section 3.1); (**b**) Black-white image of changed regions (Section 3.2); (**c**) Black-white image of suspicious landslide regions (Section 3.3); (**d**) Final result image (Section 3.4).

Results of the landslides detection in Zhouqu County are shown in Figure 8. The binary image of possible landslide areas is shown in Figure 8a. The Binary image of changed areas is shown in Figure 8b, we can find the river was incorrectly extracted, but it was removed by STCL whose result is shown in Figure 8c. Figure 8d is the result image and it shows the location and shape of the landslides.

**Figure 9:**
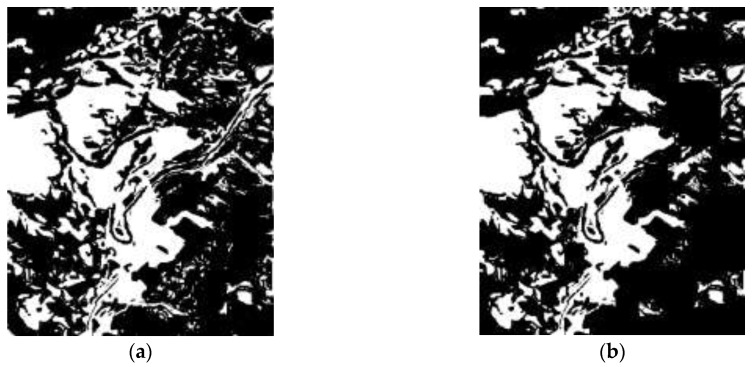

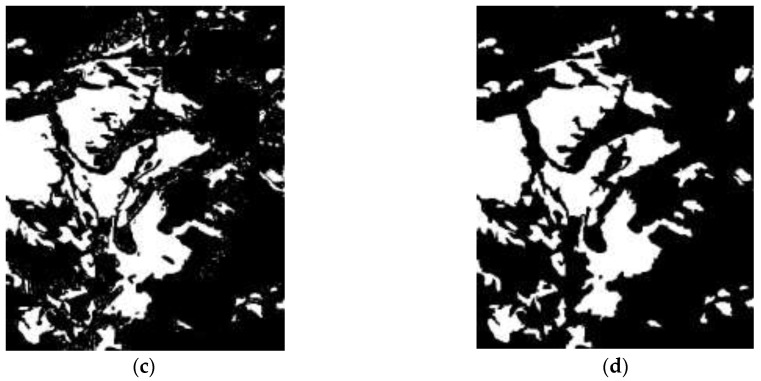
Experimental results of Beichuan County, China. (**a**) Black-white image of possible landslide regions (Section 3.1); (**b**) Black-white image of changed regions (Section 3.2); (**c**) Black-white image of suspicious landslide regions (Section 3.3); and, (**d**) Final result image (Section 3.4).

Results of the landslides detection in Beichuan County are shown in Figure 9. There are many landslides that are widely distributed and part of them are close to others, and the changed regions (Figure 9b) make up most of the image. With the help of STCL (Figure 9c) and the post process, the irrelevant regions were removed and the outline and independence of landslides were retained (Figure 9d). 

### 4.2. Accuracy Assessment

To acquire a synthetical assessment of the landslides detection method, consistency analysis between the automated detected landslides and the real ones should be done. The comparison puts the experimental areas into three classes: true positive (*TP*), false positive (*FP*) and false negative (*FN*). TPs contains the real landslide pixels which have been found by the method. FNs and FPs denotes two different error, the former represents the missing landslide pixels, and the latter represents the wrongly extracted pixels, which are not landslides.

Further, several evaluation indicators, *DP*, *QP*, and *CE* [6,14] are calculated using the following equations to implement quantitative evaluation:

(14) $$DP=100\%\times \frac{TP}{TP+FN},$$

(15) $$QP=100\%\times \frac{TP}{TP+FP+FN},$$

(16) $$CE=100\%\times \frac{FP}{TP+FP},$$

*DP* denotes the proportion of correctly detected landslides, with respect to real landslides. *QP* takes *FN* into account and measures the result synthetically. CE is error rate of detected landslides, it is the proportion of falsely detected.

The experimental results of proposed method are concluded in Table 1 based on landslide areal extent. For the case of Shenzhen, *DP* and *CE* are, respectively, 79.9% and 9.9%. Furthermore, the *QP* reaches 73.4%. For the landslides in Zhouqu County, *DP* and *CE* take the value of 70.3% and 17.6%, and the *QP* is 61.1%, less than that in Shenzhen’s landslide because of the varied topography, disperse coverage and the wispy shape of the landslide in Zhouqu County. For the landslides in Beichuan County, *DP* and *QP* reach, respectively, 88.5% and 80.1%, while *CE* is 9.6%. For the number of landslide areas, the result of accuracy assessment is shown in Table 2.

**Figure 10:**
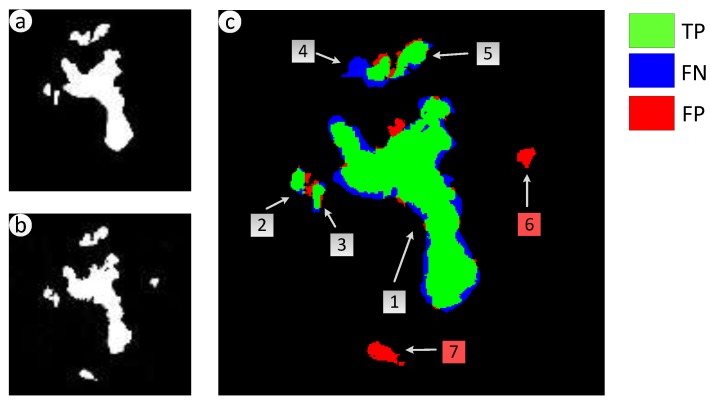
Consistency analysis of landslides in Shenzhen, China. (**a**) Real landslide regions; (**b**) Extracted Landslide regions; (**c**) Location of true positive (TP), false negative (FNs), and false positive (FPs) generated through comparison between the extracted landslides and the real ones.

**Figure 11:**
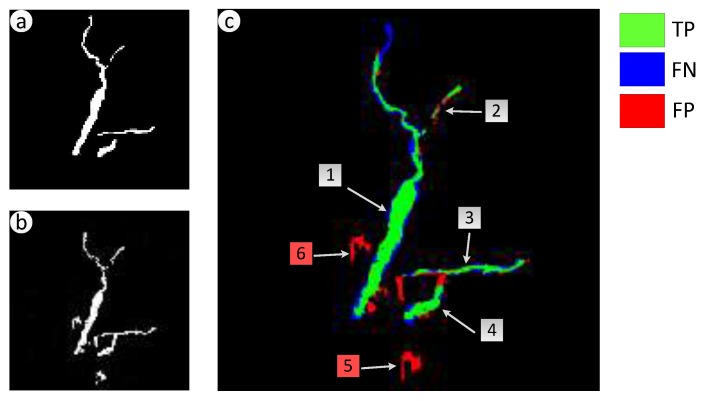
Consistency analysis of landslides in Zhouqu County, China. (**a**) Real landslide regions; (**b**) Extracted Landslide regions; (**c**) Location of TPs, FNs, and FPs generated through comparison between the extracted landslides and the real ones.

**Figure 12:**
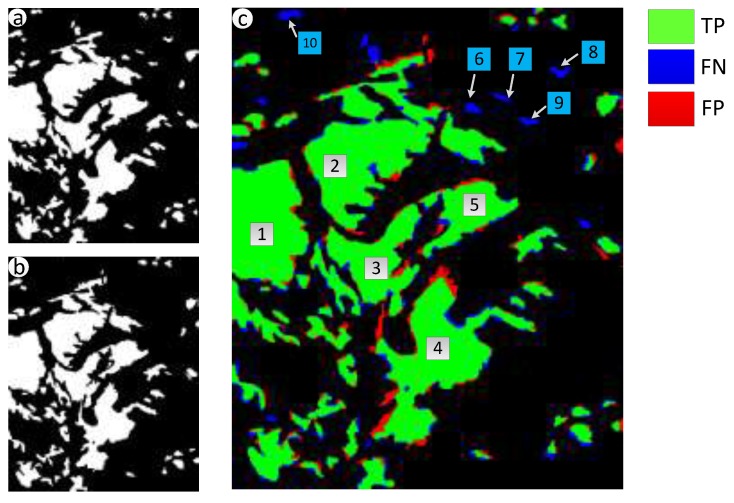
Consistency analysis of landslides in Beichuan County, China. (**a**) Real landslide regions; (**b**) Extracted Landslide regions; (**c**) Location of TPs, FNs, and FPs generated through comparison between the extracted landslides and the real ones.

To make the experimental results more intuitive, TPs, FNs, and FPs of Shenzhen, Zhouqu County, and Beichuan County are showed in Figure 10c, Figure 11c, and Figure 12c respectively, they are generated through comparison between the detected landslides and the real ones. As shown in Figure 10a,b, five landslide regions in Shenzhen have been extracted by proposed method with no one missing, achieving a recall rate of 100%. *QP* in region 1 to 5 reach 76.1%, 77.6%, 65.2%, 45.7% and 82.1% severally, region 4 only have a *QP* of 45.7% because of the cloud coverage. For the landslide in Zhouqu County, as Figure 11a,b show, the recall rate of landslide areas reaches 100%, despite some areas being wispy and tiny, and *QP* of areas 1–4 are, respectively, 70.6%, 60.0%, 58.5%, and 55.2%. Besides, there are several wrongly detected landslides areas depicted by isolated red region just like area 6–7 in Figure 10c and area 5–6 in Figure 11c, in these areas, the earth surface changes that are similar to landslide have happened artificially or naturally. For the landslides in Beichuan County, as Figure 12 shows, the method can identify large landslides well, just like landslide 1–5 in Figure 12c whose *QP* are, respectively, 95.5%, 94.0%, 92.9%, 85.3%, and 84.5%. But, there are still isolated and tiny landslides, like landslide 6–10, which have not been identified because the change degree of the surface is small.

### 4.3. Discussion

The proposed method automatically detects landslides in mountain cities based on the knowledge of the earth surface change, without other artificial spatial or spectral features, just like texture. To obtain robust and precise results in areas that cover various land surface changes caused by human or natural behaviors, regions that contain irrelevant land cover types in post-event image are filtered out in first, then change detection using DCNN is implemented for merely extracting regions where the change degree of land surface is high, therefore those scattered little regions where the land surface change are similar to landslide are removed, and the *CE* for landslides areal extent is effectively reduced.

Next, The STCL based on NDVI and PANTEX achieves fully utilizing the temporal and spatial information and the landslides-related surface change knowledge—disappearing of vegetation and built-up areas. Because the first two steps do not take account of the land covering type changing that is caused by landslides, the result image of them indicates the areas where changes have intensively taken place on large scale, the changes may be triggered by landslides or other events. With the help of STCL, regions of landslides are identified and other areas can be ruled out. Besides, the land covering models learned by STCL is based on spatial context instead of single pixel, making the changing measurement robust and stable, and can overcome the disturbance of illumination variation and salt & pepper noise.

Finally, slope parameter which is derived from post-landslide DEM is used to evaluate the detected areas and makes the results more reliable. Meanwhile, taking the size of landslides in account, tiny areas are filtered out for reducing the false alarm rate, which can also be depicted by commission error. In addition, in view of there are part of landslide areas which are without cover type changes, the change of DEM is used for detecting these landslide areas, making the shapes and outlines of the detected landslides more consistent with the ground truth.

In order to compare the proposed approach with other landslides detection approaches, two recent studies which employ the same accuracy assessment metrics have been selected. Behling et al. [17] proposed a method based on NDVI-trajectories and achieved *QP* of 50–89% and *CE* of 10–21% for landslide areal extent. Rau et al. [6] achieved *QP* of 58–81.7% and *CE* of 11–32%. In our experiment in three sites, the *QP* for landslide areal extent are between 61.1–80.1%, the *CE* for landslide areal extent is below 17.6%. For the accuracy assessment on the number of landslide areas, the *QP* and *CE* of Behling’s method is, respectively, 34.6–44.1% and 30–70%, and our approach, whose *QP* and *CE* are between 66.7–77.4% and 5.9–33.3%, performs better.

Overall, in terms of the detection accuracy, the proposed method reaches a lower commission error due to filtering the unnecessary areas based on land covering type, change degree, knowledge of land covering change, and size. The quality percentages of landslide areal extent are lower than the maximum quality percentages of Behling’s and Rau’s studies, but higher than their minimum quality percentages. For the assessment against the number of landslide areas, our approach performs better with higher quality percentages. In terms of practicality, the proposed automated approach can detect landslides in mountain cities and Behling’s method cannot identify landslides where there used to be built-up areas. Rau’s method is semiautomatic and need landslides samples which should be extracted by experts in target zone, if the detection is conduct in a new area, the proposed automated approach which need not samples and saves labor cost is more appropriate.

## 5. Conclusions

In this paper we presented an automated approach of landslides detection using multi-temporal remote sensing imagery, aiming at mountain cities. The patch-oriented change detection strategy based on DCNN model was introduced for extracting area with high change degree, and the usage of STCL makes the most of temporal and spatial information of remote sensing images for the accurate identification of landslide areas. This multi-level extraction and filtering frame achieves removing the unrelated areas and identifying the landslides gradually, and ensuring reliable detection percentage while reducing the false alarm rate.

The approach was conducted on the areas of landslide in Shenzhen, Zhouqu County and Beichuan County where both the cities and mountains existed, and the results showed that more than 70.3% landslide areal extent was extracted, meanwhile, the *CE* for landslide areal extent was below 17.6%. The experiment demonstrates that the proposed approach has a high capacity of landslide detection in mountain cities. Besides, the detection is absolutely based on the knowledge of land covering change related to landslide without extracting samples or other manual operation, thus the approach is automated and saves a lot of labor resources and time cost. However, there are still landslide areas that may not conform to the landslides-related surface change knowledge of the proposed method, like the source area of debris flow where erosion occurred, and rivers buried by landslides. Although these areas usually occupy small part of the landslides in mountain cities, more comprehensive models would be studied to solve the problem and enhance the robustness and accuracy in the next step. Since the spatial resolutions of remote sensing images in this experiment are 8 m and 30 m, in future work, the approach will be developed for images that cover larger area and has more complex natural environment. 

## Figures and Tables

**Table 1:** Accuracy statistics for landslide areal extent in three validation sites.

Validation Site	Number of TPs	Number of FPs	Number of FNs	*DP* (%)	*QP* (%)	*CE* (%)
Shenzhen	7543	832	1898	79.9	73.4	9.9
Zhouqu	2953	630	1247	70.3	61.1	17.6
Beichuan	40466	4315	5237	88.5	80.1	9.6

**Table 2:** Accuracy statistics for landslide areas in three validation sites.

Validation Site	Number of TP Areas	Number of FP Areas	Number of FN Areas	*DP* (%)	*QP* (%)	*CE* (%)
Shenzhen	5	2	0	100	71.4	28.6
Zhouqu	4	2	0	100	66.7	33.3
Beichuan	48	3	11	81	77.4	5.9
